# A generalized framework for the quantum Zeno and anti-Zeno effects in the strong coupling regime

**DOI:** 10.1038/s41598-022-23421-4

**Published:** 2022-11-04

**Authors:** Ghazi Khan, Hudaiba Soomro, Muhammad Usman Baig, Irfan Javed, Adam Zaman Chaudhry

**Affiliations:** 1 Department of Physics, Lahore University of Management Sciences (LUMS), Lahore, Pakistan; 2 Department of Mathematics and Statistics, University of New Brunswick (UNB), Frederiction, Canada

**Keywords:** Quantum physics, Statistical physics, thermodynamics and nonlinear dynamics

## Abstract

It is well known that repeated projective measurements can either slow down (the Zeno effect) or speed up (the anti-Zeno effect) quantum evolution. Until now, studies of these effects for a two-level system interacting with its environment have focused on repeatedly preparing the excited state via projective measurements. In this paper, we consider the repeated preparation of an arbitrary state of a two-level system that is interacting strongly with an environment of harmonic oscillators. To handle the strong interaction, we perform a polaron transformation and then use a perturbative approach to calculate the decay rates for the system. Upon calculating the decay rates, we discover that there is a transition in their qualitative behaviors as the state being repeatedly prepared continuously moves away from the excited state and toward a uniform superposition of the ground and excited states. Our results should be useful for the quantum control of a two-level system interacting with its environment.

## Introduction

By subjecting a quantum system to frequent and repeated projective measurements, we can slow down its temporal evolution, an effect referred to as the quantum Zeno effect (QZE)^1–24^. Contrary to this effect is the quantum anti-Zeno effect (QAZE), via which the temporal evolution of the system is accelerated due to repeated projective measurements separated by relatively longer measurement intervals^25–38^. Both these effects have garnered great interest not only due to their theoretical relevance to quantum foundations but also due to their applications to quantum technologies. For example, the QZE has shown to be a promising resource for quantum computing and quantum error correction^39,40^. The QAZE, on the other hand, has interestingly been useful in, say, accelerating chemical reactions, suggesting the possibility of quantum control of a reaction^41^.

By and large, studies of the QZE and the QAZE have focused on population decay^25–30,42–47^ and pure dephasing models^31^. A few works have gone beyond these regimes. Reference^48^, for instance, presents a general framework to calculate the effective decay rate for an arbitrary system–environment model in the weak coupling regime and finds it to be the overlap of the spectral density of the environment and a filter function that depends on the system–environment model, the measurement interval, and the measurement being performed. This approach, however, fails in the strong coupling regime where perturbation theory cannot be applied in a straightforward manner^31^. For a single two-level system coupled strongly to an environment of harmonic oscillators, Ref.^49^ makes the problem tractable by going to the polaron frame and finding that for the excited state, the decay rate very surprisingly decreases with an increase in the system–environment coupling strength. This effect is further investigated in Ref.^50^, which studies a two-level system coupled simultaneously to a weakly interacting dissipative-type environment and a strongly interacting dephasing-type one. It is found that even in the presence of both types of interactions, the strongly coupled reservoir can inhibit the influence of the weakly coupled reservoir on the central quantum system.

To date, the role of the state that is repeatedly prepared remains relatively unexplored, especially in the strong coupling regime. We emphasize that Ref.^49^ considers the relatively simple case of the repeated measurement of the excited state only. As such, it remains unanswered whether increasing the coupling strength with a strongly coupled reservoir would lead to the decay rate decreasing for any general (or arbitrary) state. This forms the basis of our investigation in this paper. We work out the decay rates for a two-level system strongly interacting with a bath of harmonic oscillators and that is repeatedly prepared via a projective measurement in an arbitrary quantum state, that is, any arbitrary linear combination of the ground and excited states. To make the problem tractable, we first go to the polaron frame, where the system–environment coupling is effectively weakened, and, thereafter, use time-dependent perturbation theory to evolve the system state and find its decay rate. Compared to the treatment in Ref.^49^, this is a far more complicated task since the polaron transformation also modifies the projection operators, thereby making the trace over the environment considerably more involved. From the decay rate, we are able to observe a stark difference when we perform projective measurements onto a uniform superposition of the excited and ground states. As we explain later, the qualitative variation of the decay rate with the system–environment coupling gets “inverted.” To describe these results, we coin the terms “*z*-type” and “*x*-type,” identifying the behavior displayed by Ref.^49^ (where increasing the coupling strength leads to smaller decay rates) as the “*z*-type” behavior and the “inverted” behavior as the “*x*-type” one. It is found that projections onto the excited or ground states on the Bloch sphere exhibit “*z*-type” behavior whereas projections onto states lying close to the equatorial plane in the Bloch sphere exhibit “*x*-type” behavior. This provides the motivation for the names “*z*-type” and “*x*-type” since it is typical to take the excited and ground states as up and down along the *z* axis, respectively, and denote their superposition as being either up or down along the *x* axis. We also investigate the transition between these *z* and *x* behaviors. Our results should be useful in the study of open quantum systems in the strong coupling regime.

## Results

### Effective decay rate for strong system–environment coupling

We start from the paradigmatic spin-boson model^51–53^ with the system–environment Hamiltonian written as (we work in dimensionless units with $\hbar = 1$ throughout)

1 $$\begin{aligned} H_{L} = \frac{\varepsilon }{2}\sigma _z + \frac{\Delta }{2} \sigma _x + \sum _{k}\omega _k b_k^{\dagger }b_k + \sigma _z\sum _{k}\left( g_k^{*} b_k + g_k b_k^{\dagger }\right) , \end{aligned}$$

where $H_{S,L} = \frac{\varepsilon }{2}\sigma _z + \frac{\Delta }{2} \sigma _x$ is the system Hamiltonian, $H_B = \sum _{k}\omega _k b_k^{\dagger }b_k$ is the environment one, and $V_L = \sigma _z\sum _{k}\left( g_k^{*} b_k + g_k b_k^{\dagger }\right)$ is the system–environment coupling. Note that *L* denotes the lab frame, $\varepsilon$ is the energy splitting of the two-level system, $\Delta$ is the tunneling amplitude, and the $\omega _k$ are the frequencies of the harmonic oscillators in the harmonic oscillator environment interacting with the system. The creation and annihilation operators of these oscillators are represented by the $b_k^\dagger$ and $b_k$, respectively. The term ‘tunneling amplitude’ for $\Delta$ is especially appropriate since it is this term that leads to transitions between the ground and excited states; if $\Delta = 0$, the excited state remains the excited state and the ground state remains the ground state. In the strong system–environment interaction regime, we cannot treat the interaction perturbatively. Moreover, the initial system–environment correlations are significant and thus cannot be neglected to write the initial state as a simple product state^54,55^. To make the problem tractable thus, we perform a polaron transformation^56–62^, which yields an effective interaction that is weak. More precisely, the transformation to the polaron frame is given by $H = U_{P}H_{L}U_P^{\dagger }$, where $U_P= e^{\frac{\chi \sigma _z}{2}}$ and $\chi = \sum _k\left( \frac{2g_k}{\omega _k}b_k^{\dagger }-\frac{2g_k^{*}}{\omega _k}b_k\right)$. We then get the transformed Hamiltonian

2 $$\begin{aligned} H= \frac{\varepsilon }{2}\sigma _z + \sum _{k}\omega _k b_k^{\dagger }b_k+ \frac{\Delta }{2}\left( \sigma _{+}e^{\chi } + \sigma _{-}e^{-\chi }\right) . \end{aligned}$$

For future convenience, we define $H_0 = \frac{\varepsilon }{2}\sigma _z + \sum _{k}\omega _k b_k^{\dagger }b_k$. Now, if $\Delta$ is taken as being small (that is, much smaller than the other energy scales such as $\varepsilon$ and $g_k$), the system and environment interact effectively weakly in the polaron frame despite interacting strongly in the lab frame. Let $\left| 0\right\rangle$ represent the excited state of our two-level system and $\left| 1\right\rangle$ its ground state. Then, writing an arbitrary initial state of the two-level system as $\left| \psi \right\rangle = \zeta _{1} \left| 0\right\rangle + \zeta _{2} \left| 1\right\rangle$ with $\zeta _1 = \cos {\left( \frac{\theta }{2}\right) }$ and $\zeta _2 = e^{i\phi }\sin {\left( \frac{\theta }{2}\right) }$, where $\theta$ and $\phi$ are the standard angles on the Bloch sphere, we find the time-evolved density matrix by means of time-dependent perturbation theory. It is important to note that while the initial system–environment state cannot simply be taken as a simple uncorrelated product state in the lab frame^54,55^, we can do so in the polaron frame since the system and its environment interact effectively weakly in it. We subsequently perform repeated measurements after time intervals of duration $\tau$ to see if the system state is still $\left| \psi \right\rangle$. The survival probability at time $\tau$ is $s(\tau )= \hbox {Tr}_{S,B}\left\{ {P_{\psi }\rho (\tau )}\right\}$, where $\rho (\tau )$ is the combined density matrix of the system and the environment at time $t=\tau$ in the polaron frame just before a projective measurement, $P_{\psi }= U_P\left| {\psi }\right\rangle \left\langle {\psi }\right| U_P^{\dagger }$, and *S* and *B* denote traces over the system and the bath of harmonic oscillators respectively. The survival probability can then be written as

3 $$\begin{aligned} s(\tau ) = \hbox {Tr}_{S,B}\left\{ P_{\psi }e^{-iH\tau } P_{\psi }\frac{e^{-\beta H_{0}}}{Z} P_{\psi }e^{iH\tau }\right\} \end{aligned}$$

with $Z = \text {Tr}_{S, B}\{P_{\Psi }e^{-i H\tau }P_{\Psi }\}$ being a normalization factor and $\beta$ representing the inverse temperature. Until now, the treatment of the survival probability is completely general. Ref.^49^ proceeds by considering only the simplest case where $[U_P, \left| \psi \right\rangle \left\langle \psi \right| ] = 0$, which means that the polaron transformation leaves the projector $\left| \psi \right\rangle \left\langle \psi \right|$ untouched. This is only true for the states $\left| 0\right\rangle$ and $\left| 1\right\rangle$. The assumption that $[U_P, \left| \psi \right\rangle \left\langle \psi \right| ] = 0$ greatly simplifies the subsequent calculation since the projection operator $P_\psi$ in the polaron frame contains no environment operators. In the more general case of $\left| \psi \right\rangle = \zeta _{1} \left| 0\right\rangle + \zeta _{2} \left| 1\right\rangle$ that we are considering in the current paper with arbitrary $\zeta _1$ and $\zeta _2$, the presence of the additional environment operators in $P_\psi$ makes the calculation far more complicated. The details of this calculation are presented in the “Methods” section, and the rather extensive general expression is given in the Supplementary information. In order to digest this and to introduce the environment functions that appear, it is useful to first consider the limited case with the initial state $\left| \psi \right\rangle = \left| 0\right\rangle$ and the projector $P_\psi = \left| 0\right\rangle \left\langle 0\right|$. For this case, our general expression reduces to

4 $$\begin{aligned} s(\tau ) = 1-2{\text{Re}}{\frac{\Delta ^2}{4}\int _{0}^{\tau } dt_{1}\int _{0}^{t_{1}}dt_{2}\left| \zeta _{1}\right| ^2 e^{-i\varepsilon t_1} e^{i\varepsilon t_2}C(t_{2}-t_{1})}, \end{aligned}$$

meaning that we reproduce the expression given in Ref.^49^. Here, $C(t_{2}-t_{1})$ is the environment correlation function given by $C(t_{2}-t_{1})= e^{-\Phi _{C}^{*}(t_2 - t_1)}$, $\Phi _{C}(t)= \Phi _R(t) - i\Phi _I(t)$ with $\Phi _{R}= 4\int _{0}^{\infty }d\omega J(\omega )\frac{1-\cos {\omega t}}{\omega ^2}\coth ({\frac{\beta \omega }{2}})$ and $\Phi _{I}=4\int _{0}^{\infty }d\omega J(\omega )\frac{\sin {\omega t}}{\omega ^2}$. The environment spectral density $J(\omega )$ has been introduced as $\sum _{k}\left| g_k\right| ^2(\cdots ) \rightarrow \int _{0}^{\infty }d\omega J(\omega )(\cdots )$. Since the system–environment coupling in the polaron frame is weak, we can neglect the accumulation of correlations between the system and the environment and write the survival probability at time $t=N\tau$, or $s(t=N\tau )$, as $[s(t)]^N$, where *N* denotes the number of measurements performed after time $t=0$. Now, we may write $s(t=N\tau ) = e^{-\Gamma (\tau )N\tau }$ to define an effective decay rate $\Gamma (\tau )$ for our quantum state. It follows that $\Gamma (\tau )= -\frac{1}{\tau }\ln {s(\tau )}$. Expanding $\ln (s(\tau ))$ up to second order in $\Delta$, we see that the decay rate works out to be $\frac{1-s(\tau )}{\tau }$. Furthermore, in order to numerically investigate how the decay rate varies with the measurement interval $\tau$, we model the spectral density as $J(\omega )= G\omega ^{s}\omega _{c}^{1-s}e^{-\omega /\omega _c}$, where *G* is a dimensionless parameter characterizing the strength of the system–environment coupling, $\omega _c$ is the cut-off frequency, and *s* is the so-called Ohmicity parameter. Throughout, we present results for a super-Ohmic environment with $s=2$. For this case, we get $\Phi _{R}= 4G\left( 1-\frac{1}{1+\omega _c^2 t^2}\right)$ and $\Phi _{I}= \frac{4Gt}{\omega _c\left( \frac{1}{\omega _c^2}+t^2\right) }$, and we choose to work at zero temperature for the sake of simplicity. We thus obtain

5 $$\begin{aligned} \Gamma (\tau )= \frac{\Delta ^2}{2\tau }{\text{Re}}\left\{{\int _{0}^{\tau } dt_{1}\int _{0}^{t_{1}}dt_{2}e^{-i\varepsilon (t_1-t_2)} e^{-4G\left( 1-\frac{1}{1+\omega _c^2 (t_2-t_1)^2}\right) }e^{-i\frac{4G(t_2-t_1)}{\omega _c\left[ \frac{1}{\omega _c^2}+(t_2-t_1)^2\right] }}}\right\}, \end{aligned}$$

and note that the same decay rate is found if we repeatedly prepare the ground state instead (that is, we set $\zeta _1 = 0$ and $\zeta _2 = 1$ instead in our general expression).

**Figure 1:**
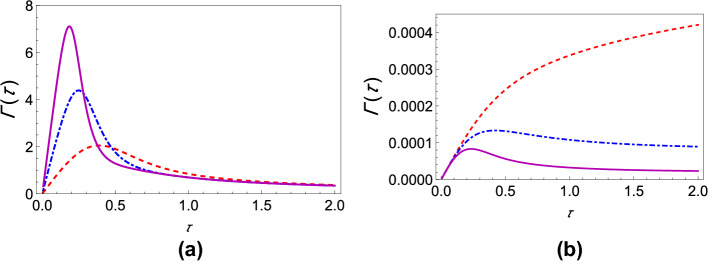
Variation of the effective decay rate with system–environment coupling strength. (**a**) Graph of $\Gamma (\tau )$ (at zero temperature) as a function of $\tau$ with $G=1$ (dashed, red curve), $G=2$ (dot-dashed, blue curve), and $G=3$ (solid magenta curve). The initial state is $\cos \left( \theta /2\right) \left| 0\right\rangle +e^{\iota \phi }\sin \left( \theta /2\right) \left| 1\right\rangle$ with $\theta = \pi /2$ and $\phi = 0$, but any other $\phi$ would give the same results here. We have also used a super-Ohmic environment ($s=2$) with $\omega _c = 1$, $\varepsilon =1$, and $\Delta = 0.05$. (**b**) Graph of $\Gamma (\tau )$ (at zero temperature) as a function of $\tau$ with $G=1$ (dashed, red curve), $G=2$ (dot-dashed, blue curve), and $G=3$ (solid magenta curve). The initial state is given by $\theta = 0$ and $\pi = 0$. We have used a super-Ohmic environment ($s=2$) with $\omega _c = 1$, $\varepsilon =1$, and $\Delta = 0.05$.

**Figure 2:**
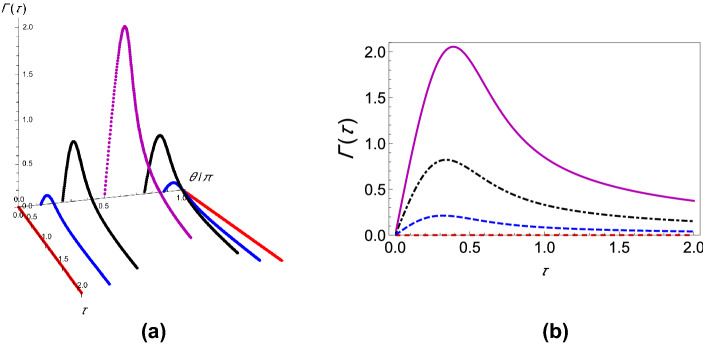
Variation of the effective decay rates with the initial state of the two-level system. (**a**) The effective decay rate $\Gamma (\tau )$ (at zero temperature) as a function of $\tau$ for different initial states. $\theta$ takes values from 0 through $\pi$, but $\phi$ is kept 0 for the sake of simplicity. (**b**) $\Gamma (\tau )$-$\tau$ cross-sections of (**a**) for $\theta =0$ (dashed, red curve), $\theta =\pi /8$ (dashed, blue curve), $\theta =\pi /4$ (dot-dashed, black curve), and $\theta = \pi /2$ (solid magenta curve). We have used a super-Ohmic environment ($s=2$) with $G = 1$, $\omega _c = 1$, $\varepsilon =1$, and $\Delta = 0.05$.

Now, consider the repeated preparation of an arbitrary quantum state of the two-level system. As noted before, for this case, the projection operator, in the polaron frame, contains environment operators. Consequently, taking the trace over the environment is much more complicated. Here, we present the results for the case $\zeta _{1} = \frac{1}{\sqrt{2}}$ and $\zeta _{2} = \frac{1}{\sqrt{2}}$, that is, $\left| \psi \right\rangle = \frac{1}{\sqrt{2}}\left| 0\right\rangle +\frac{1}{\sqrt{2}}\left| 1\right\rangle$, although we emphasize that our developed formalism allows us to work out the effective decay rate for any arbitrary state—see the “Methods” section and the supplementary information. Working at zero temperature again, we find the effective decay rate to be

6 $$\begin{aligned} \begin{aligned} \Gamma (\tau )=&\,\frac{1}{\tau }\bigg \{1-\frac{1}{4}\bigg [2e^{\beta \varepsilon /2}+ 2{\text{Re}}\bigg \{e^{i\varepsilon \tau }e^{\beta \varepsilon /2}e^{-\Phi _{C}(\tau )}\bigg \}\\&\,\ \ \ \ \ +2{\text{Re}}\bigg \{i\frac{\Delta }{2}\int _{0}^{\tau }dt_1 \bigg (e^{-i\varepsilon t_1}e^{\beta \varepsilon /2}e^{-\Phi _{C}^{*}(t_1)}+e^{i\varepsilon t_1}e^{\beta \varepsilon /2}e^{-\Phi _{C}(t_1)}+e^{i\varepsilon \tau }e^{-i\varepsilon t_1}e^{\beta \varepsilon /2}e^{-\Phi _{C}^{*}(t_1-\tau )}\\&\,\ \ \ \ \ +e^{-i\varepsilon \tau } e^{i\varepsilon t_1}e^{\beta \varepsilon /2}e^{-\Phi _{C}^{*}(t_1-\tau )}e^{-2i\Phi _{I}(\tau )}e^{2i\Phi _{I}(t_1)}\bigg )\bigg \}\\&\,\ \ \ \ \ -2{\text{Re}}\bigg \{\frac{\Delta ^2}{4}\int _{0}^{\tau }dt_1\int _{0}^{t_1}dt_2 \bigg ( e^{-i\varepsilon t_1}e^{i\varepsilon t_2}e^{\beta \varepsilon /2}e^{-\Phi _{C}^{*}(t_2-t_1)}e^{2i\Phi _{I}(t_2)}e^{-2i\Phi _{I}(t_1)}+e^{i\varepsilon t_1}e^{-i\varepsilon t_2}e^{\beta \varepsilon /2}e^{\Phi _{C}^{*}(t_2-t_1)}\\&\,\ \ \ \ \ +e^{i\varepsilon \tau }e^{-i\varepsilon t_1}e^{i\varepsilon t_2}e^{\beta \varepsilon /2}W' e^{i\Phi _{I}(t_2)}e^{-i\Phi _{I}(t_1)}e^{i\Phi _{I}(t)}+e^{-i\varepsilon \tau }e^{i\varepsilon t_1}e^{-i\varepsilon t_2}e^{\beta \varepsilon /2}W' e^{-i\Phi _{I}(t_2)}e^{i\Phi _{I}(t_1)}e^{-i\Phi _{I}(\tau )}\bigg )\bigg \}\\&\,\ \ \ \ \ + \frac{\Delta ^2}{4}\int _{0}^{\tau }dt_1\int _{0}^{\tau }dt_2\bigg (e^{i\varepsilon t_1}e^{-i\varepsilon t_2}e^{\beta \varepsilon /2}e^{-\Phi _{C}^{*}(t_2-t_1)}+ e^{-i\varepsilon t_1}e^{i\varepsilon t_2}e^{\beta \varepsilon /2} e^{-\Phi _{C}^{*}(t_2-t_1)}e^{2i\Phi _{I}(t_2)}e^{-2i\Phi _{I}(t_1)}\\&\,\ \ \ \ \ +e^{i\varepsilon \tau }e^{-i\varepsilon t_1}e^{-i\varepsilon t_2}e^{\beta \varepsilon /2}W e^{-i\Phi _{I}(t_2)}e^{-i\Phi _{I}(t_1)}e^{i\Phi _{I}(\tau )}+e^{-i\varepsilon \tau }e^{i\varepsilon t_1}e^{i\varepsilon t_2}e^{\beta \varepsilon /2}We^{i\Phi _{I}(t_2)}e^{i\Phi _{I}(t_1)}e^{i\Phi _{I}(\tau )} \bigg )\bigg ]\bigg \}, \end{aligned} \end{aligned}$$

where *W* and $W'$ comprise further bath correlation terms and are given in the supplementary information. In Eqs. (5) and (6), what we have essentially found are the decay rates for initial states corresponding to two extremes characterized by the positions of the states on the Bloch sphere. Whereas Eq. (5) gives the decay rate for the state $\left| \psi \right\rangle$ on a pole of the Bloch sphere, Eq. (6) does so for a state that is the farthest from the poles. Note that the effective decay rate is independent of $\phi$ when $\theta$ is chosen to be $\frac{\pi }{2}$ in $\left| \psi \right\rangle = \cos \left( \frac{\theta }{2}\right) \left| 0\right\rangle +e^{\iota \phi }\sin \left( \frac{\theta }{2}\right) \left| 1\right\rangle$, so Eq. (6) may be seen as catering to all states lying in the equatorial plane of the Bloch sphere. The states $\left| 0\right\rangle$ and $\frac{1}{\sqrt{2}}\left| 0\right\rangle +\frac{1}{\sqrt{2}}\left| 1\right\rangle$ were chosen as simple representatives of the poles and the equatorial plane, respectively. These regions comprise the said extremes since we find that the respective variations of the effective decay rate with the system–environment coupling in these regions are markedly opposite. While increasing the coupling strength leads to a decrease in the decay rate for states on the poles, the opposite occurs when the coupling strength is increased for states lying in the equatorial plane. To make this claim concrete, we work out the integrals in Eqs. (5) and (6) numerically and plot the effective decay rate $\Gamma (\tau )$ in Fig. 1 for various system–environment coupling strengths. As Fig. 1a clearly shows, increasing the coupling strength effects a general increase in the decay rate if the initial state is $\frac{1}{\sqrt{2}}\left| 0\right\rangle +\frac{1}{\sqrt{2}}\left| 1\right\rangle$. Precisely the opposite is seen in Fig. 1b; that is, increasing the system–environment coupling decreases the effective decay rate when the initial state is $\left| 0\right\rangle$. It might therefore be said that uniform superpositions essentially “invert” the inhibiting effect that an increase in the coupling strength has on the decay rate in Fig. 1b. The behavior of $\Gamma (\tau )$ as a function of $\tau$ also allows us to identify the Zeno and anti-Zeno regimes. The Zeno regime is the region where decreasing $\tau$ leads to a decrease in $\Gamma (\tau )$. The anti-Zeno regime, alternatively, happens to be the region where decreasing $\tau$ leads to an increase in $\Gamma (\tau )$^25,31,37,43,46,48^. With these criteria, whereas we observe only the QZE for $G=1$ in Fig. 1b, we also see the QAZE for $G=2$ and $G=3$. In Fig. 1a, however, we see both the QZE and the QAZE for all the coupling strengths shown. Increasing *G* thus bears forth a significant qualitative change in the QZE/QAZE behavior of the central quantum system if the initial state is on the poles of the Bloch sphere, but the same is not true for uniform superpositions. This also tells us that the aforementioned two regions on the Bloch sphere are characterized by significantly different variations of the decay rate with the system–environment interaction. Finally, it is worth noting that for uniform superpositions, the decay rates are generally much higher than the ones corresponding to the ground or excited states (see Fig. 1a,b). This result makes sense because in the strong coupling regime, the system–environment coupling acts as a protection for its eigenstates, meaning that the eigenstates of the interaction term actually benefit from an increased coupling with the environment in that they remain alive for longer times^49^. This protection, however, is lost as we move away from a pole on the Bloch sphere and toward the equatorial plane as is apparent in Fig. 2, where we plot the decay rates against $\tau$ for varying polar angles.

**Figure 3:**
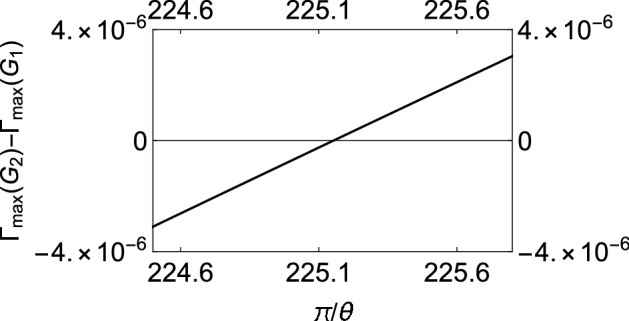
Difference between the maxima of the effective decay rates corresponding to $G_1$ and $G_2$ against $\pi /\theta$.

**Figure 4:**
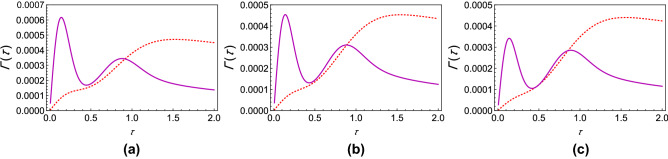
Transitory behavior in the effective decay rates. (**a**) Graph of $\Gamma (\tau )$ (at zero temperature) with the initial state corresponding to $\theta =\pi /200$ for $G=1$ (solid magenta curve) and $G=3$ (dashed, red curve). (**b**) Same as (**a**) with the initial state corresponding to $\theta _c = \pi /225$, showing critical behavior. (**c**) Same as (**a**) except that $\theta =\pi /250.$

If $\Gamma (\tau )$ is plotted against $\tau$ for different values of $\theta$, it is found that for any coupling strength, all the initial states have decay rates with one maximum. If we now assume two different coupling strengths, say, $G_1$ and $G_2$, and we consider $G_2>G_1$ without loss of generality, we notice that the decay rates exhibit either “*z*-type” or “*x*-type” behavior, depending on the initial state of the quantum system. For states we term as having “*z*-type” behavior, the maximum of $\Gamma (\tau )$ corresponding to $G_1$ is greater as is characteristic of the state $\left| 0\right\rangle$ in Fig. 1b. Similarly, we term states as showing “*x*-type” behavior if the maximum of $\Gamma (\tau )$ corresponding to $G_1$ is smaller. Hence, for any $G_1$ and $G_2$, there has to exist a value of the angle $\theta$ at which we see a transition between these two behaviors. To show the existence of this critical value of $\theta$, which we label as $\theta _c$, we plot the difference between the respective maxima of decay rates corresponding to $G_1$ and $G_2$ against $\theta$ (see Fig. 3) and find the value of $\theta$ such that this difference becomes approximately zero. To show that $\theta _c$ is actually the said critical value of $\theta$, we plot the decay rates against $\tau$ for values of $\theta$ less than $\theta _c$, equal to $\theta _c$, and greater than $\theta _c$ as illustrated in Fig. 4. It is clear that when $\theta = \theta _c$ (approximately $\pi / 225$ for the case chosen), the peaks of the curves corresponding to $G_1$ and $G_2$ are at the same height above the $\tau$ axis. When $\theta < \theta _c$, the peak for $G_2$ wins, showing that the “*x*-type” behavior dominates, and when $\theta > \theta _c$, the peak for $G_1$ wins, showing that the “*z*-type” behavior dominates. It is interesting to note that $\theta _{c}$ is $\phi$ dependent. While we have presented our analysis with $\phi = 0$, we have found that it is always possible to find $\theta _{c}$ regardless of the value of $\phi$.

### Modified decay rates for strong and weak system–environment coupling

**Figure 5:**
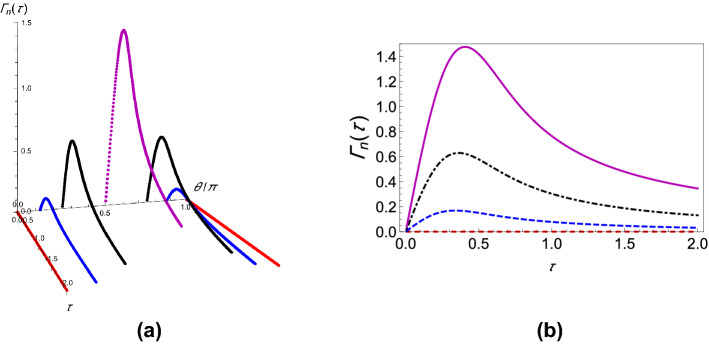
Variation of the modified decay rates with the initial state of the two-level system. (**a**) The effective modified decay rate $\Gamma _n(\tau )$ as a function of $\tau$ for different initial states. (**b**) Here we show $\Gamma _n(\tau )-\tau$ cross-sections of (**a**). The parameters used in (**a**) and (**b**) are the same as Fig. 2, except that we are now plotting the modified decay rates against the measurement interval.

**Figure 6:**
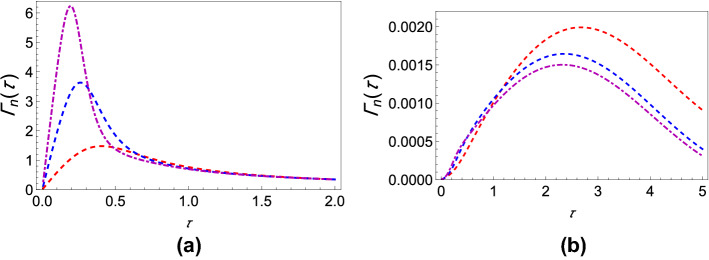
Variation of the modified decay rate with the system–environment coupling strength. (**a**) Graph of $\Gamma _n(\tau )$ as a function of the measurement interval $\tau$ for $\theta = \pi /2$ and $\phi = 0$ with different environment coupling strengths. (**b**) Graph of $\Gamma _n(\tau )$ as a function of $\tau$ for $\theta = 0$ and $\phi = 0$ with different coupling strengths. The parameters used in (**a**) and (**b**) are the same as Fig. 1, except that we are now showing the modified decay rates.

In investigating the effect of changing the initial state on the QZE and the QAZE, we have used the complete Hamiltonian so far. This means that the evolution of the system state depends on the system Hamiltonian as well as the system–environment interaction. However, if we intend to study solely the effect of the dephasing reservoir on the QZE and the QAZE via its interaction with the system, we would like to remove the evolution due to the system Hamiltonian. We can do so by performing, just before each projective measurement, a reverse unitary time evolution due to the system Hamiltonian on the fully time-evolved density matrix as has also been done in Refs.^31,48,49,63^—such a reverse unitary time evolution can be realized by applying suitable control pulses to the central two-level system. The survival probability then becomes

7 $$\begin{aligned} s(\tau )=Tr_{S,B}\left\{ P_{\psi }U_{S,I}^{\dagger }(\tau )U_{S,0}^{\dagger }(\tau )U_0(\tau ) U_I(\tau ) P_{\psi }\frac{e^{-\beta H}}{Z} P_{\psi } U_I^{\dagger }(\tau )U_0^{\dagger }(\tau )U_{S,0}(\tau )U_{S,I}(\tau )\right\} , \end{aligned}$$

where $U_{S,0}(\tau )= e^{-iH_{S}\tau }$ and $H_{S}=\frac{\varepsilon }{2}\sigma _z + \frac{\Delta }{2}\sigma _x$. As before, we can simplify this further by operating in the polaron frame (see the “Methods” section for details). This procedure yields the decay rate

8 $$\begin{aligned} \Gamma _{n}(\tau ) = \Gamma (\tau ) + \Gamma _{\text {mod}}(\tau ). \end{aligned}$$

Equation (8) shows that upon removing the system evolution, the decay rate works out to contain both the earlier found effective decay rate and some additional terms represented by $\Gamma _{\text {mod}}(\tau )$. This follows from the further application of the perturbative approach. We present in the supplementary information a general expression for the modified decay rate for an arbitrary state $\left| \psi \right\rangle$. Using this expression, we show the behavior of the modified decay rate as a function of the measurement interval for different states in Fig. 5. We again find that the decay rate increases as we move toward the circle of uniform superpositions on the Bloch sphere. Moreover, Fig. 6a shows that increasing the system–environment coupling strength generally increases the decay rate for $\theta = \frac{\pi }{2}$. As such, the removal of the system evolution does not change the qualitative behavior of the decay rates in any significant way, and we can confidently say that the primary contribution to the decay rate comes from the system–environment interaction. We arrive at a similar conclusion upon plotting the decay rate corresponding to the initial state $\left| 0\right\rangle$ in Fig. 6b, that is, the qualitative behavior remains the same as before and increasing the coupling strength now generally decreases the decay rate. We again emphasize that we could have chosen any states other than $\frac{1}{\sqrt{2}}\left| 0\right\rangle +\frac{1}{\sqrt{2}}\left| 1\right\rangle$ and $\left| 0\right\rangle$ since our general expression for the modified decay rate works for any arbitrary state.

**Figure 7:**
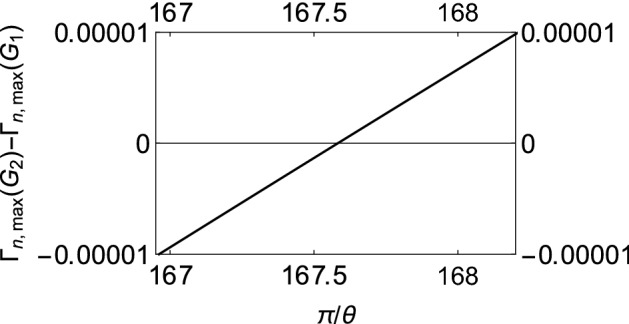
Difference between the maxima of the modified decay rates corresponding to $G_1$ and $G_2$ against $\pi /\theta$.

**Figure 8:**
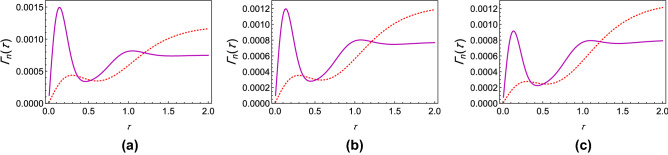
Transitory behavior in the modified decay rates. All is the same as in Fig. 4 except that we have modified decay rates, and the $\theta$ values for (**a**), (**b**), and (**c**) are $\theta =\pi /150$, $\theta _c = \pi /167$, and $\theta =\pi /190$, respectively.

To complete our analysis, we can, as before, numerically sample through decay rates corresponding to different points on the Bloch sphere to identify a transitory stage, or $\theta _c$, and find similar transitions as found before (see Fig. 7). As we move toward the poles (see Fig. 8a–c), we observe the shift from the “*x*-type” behavior to the “*z*-type” behavior.

## Discussion

To conclude, we have extended the investigation of the QZE and the QAZE for a two-level system interacting strongly with a harmonic oscillator bath by presenting a general framework independent of the initial state chosen. We started off by transforming to the polaron frame wherein the perturbative approach was used to make the problem tractable. From there on, we proceeded to finding the effective and modified decay rates, obtaining the latter after removing the system evolution so that the role of the environment alone may be studied. We found that the effective and modified decay rates display the same qualitative behavior; this attests to the dominant contribution of the reservoir to the decay rates. Having set up the methodology, we continued to investigate the effect of changing the initial state on the QZE and the QAZE, allowing ourselves to identify the “*z*-type” and the “*x*-type” behaviors. We were also able to locate critical angles about which transition between these behaviors is manifested. All these insights can be helpful for quantum control of two-level systems that are strongly interacting with a harmonic oscillator environment.

## Methods

### Polaron transformation

Here, we present the polaron transformation for the spin-boson Hamiltonian. The transformation is given by the unitary operator $U_P= e^{\frac{\chi \sigma _z}{2}}$ such that $H= e^{\frac{\chi \sigma _z}{2}}H_{L}e^{-\frac{\chi \sigma _z}{2}}$, where $\chi = \sum _k\left( \frac{2g_k}{\omega _k}b_k^{\dagger }-\frac{2g_k^{*}}{\omega _k}b_k\right)$. Making use of $e^{\theta X}Ye^{-\theta X} = Y+ \theta [X, Y] + \frac{\theta ^2}{2!} \left[ X,\left[ X,Y\right] \right] +\cdots$, we first evaluate $\left[ \frac{\chi \sigma _z}{2},\sum _k \omega _k b_k^{\dagger }b_k\right]$, $\left[ \frac{\chi \sigma _z}{2},\sigma _z\sum _k\left( g_k^{*}b_k + g_k b_k^{\dagger }\right) \right]$, and all the higher-order commutators. We find that $\left[ \frac{\chi \sigma _z}{2},\sum _k \omega _k b_k^{\dagger }b_k\right] =-\sigma _z\sum _k\left( g_k b_k^{\dagger } + g_k^{*}b_k\right)$ and $\left[ \frac{\chi \sigma _z}{2},\left[ \frac{\chi \sigma _z}{2},\sum _k \omega _k b_k^{\dagger }b_k\right] \right] = \left[ \frac{\chi \sigma _z}{2},\sigma _z\sum _k\left( g_k^{*}b_k + g_k b_k^{\dagger }\right) \right] =-2\sum _k\frac{\left| g_k\right| ^2}{\omega _k}$. Since the latter is only a constant, the higher-order commutators are zero. Moreover, since the tunneling term could be written in the form $\frac{\Delta }{2}\sigma _x = \frac{\Delta }{2}(\sigma _+ + \sigma _-)$ and $\left[ \frac{\chi \sigma _z}{2},\sigma _+\right] = \chi \sigma _{+}$ while $\left[ \frac{\chi \sigma _z}{2},\sigma _-\right] = -\chi \sigma _{-}$, the tunneling term in the polaron frame is $\frac{\Delta }{2}(\sigma _+ e^{\chi } + \sigma _-e^{-\chi })$ . Using these commutators, we get $e^{\chi \sigma _z/2}\bigg [\frac{\varepsilon }{2}\sigma _z +\frac{\Delta }{2}\sigma _x + \sum _k\omega _k b_k^{\dagger }b_k + \sigma _z\sum _k\left( g_k^{*} b_k+ g_k b_k^{\dagger }\right) \bigg ]e^{-\chi \sigma _z/2} = \frac{\varepsilon }{2}\sigma _z + \sum _k\omega _k b_k^{\dagger }b_k + \frac{\Delta }{2}(\sigma _+ e^{\chi } + \sigma _-e^{-\chi }).$ This gives the polaron transformed Hamiltonian.

### Effective decay rate for a strongly interacting environment

We now describe the procedure for deriving the decay rate from the survival probability stated in Eq. (3). To do so, we first work out the time-evolved density matrix of the composite system, that is, $\rho (\tau )=e^{-iH\tau } P_{\psi }\frac{e^{-\beta H_{0}}}{Z} P_{\psi }e^{iH\tau }$. Since we are in the polaron frame and we take $\Delta$ as being small, the effective system–environment interaction may be treated perturbatively. Now, $\rho (\tau )= U_{0}(\tau )U_{I}(\tau )\rho (0)U_{I}^{\dagger }(\tau )U_{0}^{\dagger }(\tau )$, where $U_0(\tau )$ is the unitary time-evolution operator corresponding to the system Hamiltonian $H_S=\frac{\varepsilon }{2}\sigma _z$ and the environment Hamiltonian $H_B =\sum _k\omega _k b_k^{\dagger }b_k$ whereas $U_I(\tau )$ is the unitary evolution due to the system–environment interaction. The survival probability thus becomes $s(\tau )=\hbox {Tr}_{S,B}\left\{ P_{\psi }U_{0}(\tau )U_{I}(\tau )P_{\psi }\frac{e^{-\beta H_{0}}}{Z}P_{\psi }U_{I}^{\dagger }(\tau )U_{0}^{\dagger }(\tau )\right\} .$ Using cyclic invariance, we absorb the system time evolution into the projector $P_\psi$ and evolve it to $P_{\psi }(\tau )= U_{0}^{\dagger }(\tau )P_{\psi }U_{0}(\tau )$, thereby getting $P_{\psi }(\tau )=\left| \zeta _{1}\right| ^2 \left| {0}\right\rangle \left\langle {0}\right| + \zeta _{1}\zeta _{2}^{*}e^{\chi (\tau )}e^{-i\varepsilon \tau }\left| {0}\right\rangle \left\langle {1}\right| + \zeta _{2}\zeta _{1}^{*}e^{-\chi (\tau )}e^{-i\varepsilon \tau }\left| {1}\right\rangle \left\langle {0}\right| + \left| \zeta _{2}\right| ^{2}\left| {1}\right\rangle \left\langle {1}\right|$. Now, we proceed to find $U_{I}(\tau )P_{\psi }\frac{e^{-\beta H_{0}}}{Z}P_{\psi }U_{I}^{\dagger }(\tau )$. Recalling that the interaction Hamiltonian is $H_{I}=\frac{\Delta }{2}(\sigma _{+}e^{\chi } + \sigma _{-}e^{-\chi })$ in the polaron frame and writing $V_{I}(t)=e^{iH_{0} t}H_{I}(t)e^{-iH_{0} t}$, we get $V_{I}(t)= \frac{\Delta }{2}\sum _{\mu }(\widetilde{F}_{\mu }(t)\otimes \widetilde{B}_{\mu }(t))$, where $\widetilde{F}_0(t)= \sigma _{-}e^{-i\varepsilon t}$, $\widetilde{F}_1( t)= \sigma _{+}e^{i\varepsilon t}$, $\widetilde{B}_0(t)= e^{\chi (t)}$, and $\widetilde{B}_1(t)= e^{-\chi (t)}$. This gives $U_{I}(\tau )= 1 - i\int _0^{\tau }dt_1 V_I(t_1) - \int _0^{{\tau }}\int _0^{t_{1}}dt_{1}dt_{2} V_I(t_{1})V_I(t_{2})+\cdots$. Defining $A_1(\tau ) = -i\int _0^{\tau }dt_1 V_I(t_1)$ and $A_2(\tau )=-\int _0^{{\tau }}\int _0^{t_{1}}dt_{1}dt_{2} V_I(t_{1})V_I(t_{2})$, we find that $\rho (\tau )$ up to the second order is

9 $$\begin{aligned} \rho (\tau ) = \rho (0) + A_1(\tau )\rho (0) + A_2(\tau )\rho (0) + \rho (0) A_1^{\dagger }(\tau ) +\rho (0) A_2^{\dagger }(\tau ) + A_1(\tau )\rho (0) A_1^{\dagger }(\tau ). \end{aligned}$$

It should be noted that $\rho (0)$ as given in the Results section may be written as $P_{\psi }e^{-\beta H_{0}}P_{\psi }/Z = \sum _{ijn} M_{ij}C_{ij}^{n}E_{ij}^{n}/Z$, where *i*, *j*, and *n* could be either 0 or 1. $M_{ij}=\left| {i}\right\rangle \left\langle {j}\right|$, and the $C_{ij}^{n}$ and the $E_{ij}^{n}$ are given by the following tables:

**Table 1:** The symbols $C^{n}_{ij}$.

n/ij	00	01	10	11
0	$\left| \zeta _1\right| ^4 e^{-\beta \varepsilon /2}$	$\left| \zeta _1\right| ^2\zeta _1 \zeta _2^{*}e^{-\beta \varepsilon /2}$	$\left| \zeta _1\right| ^2\zeta _2 \zeta _1^{*}e^{-\beta \varepsilon /2}$	$\left| \zeta _1 \zeta _2\right| ^2e^{-\beta \varepsilon /2}$
1	$\left| \zeta _1 \zeta _2\right| ^2 e^{\beta \varepsilon /2}$	$\left| \zeta _2\right| ^2\zeta _1 \zeta _2^{*}e^{\beta \varepsilon /2}$	$\left| \zeta _2\right| ^2\zeta _2 \zeta _1^{*}e^{\beta \varepsilon /2}$	$\left| \zeta _2\right| ^4e^{\beta \varepsilon /2}$

**Table 2:** The symbols $E^{n}_{ij}$. Here, $H_B = \sum _k \omega _k b_k^\dagger b_k$.

n/ij	00	01	10	11
0	$e^{-\beta H_B}$	$e^{-\beta H_B}e^{\chi }$	$e^{\chi }e^{-\beta H_B}$	$e^{-\chi }e^{-\beta H_B}e^{\chi }$
1	$e^{\chi }e^{-\beta H_B}e^{-\chi }$	$e^{\chi }e^{-\beta H_B}$	$e^{-\beta H_B}e^{-\chi }$	$e^{-\beta H_B}$

Using Tables 1 and 2, we find it easy to see that Eq. (9) may be recast as $\rho (\tau )=\sum _{i=1}^{6} T_i$, where $T_1 = \sum _{ijn}M_{ij}C_{ij}^{n}E_{ij}^{n}$, $T_2 = T_1 A_1^{\dagger }$, $T_3 = T_{1}A_{2}^{\dagger }$, $T_4 = A_{1}T_{1}$, $T_5= A_{1}T_{1}A_1^{\dagger }$, and $T_6 = A_{2}T_{1}$. Having found $\rho (\tau )$ and $P_{\psi }(\tau )$, we have $s(\tau )= \hbox {Tr}_{S,B}\{{P_{\psi }(\tau )\rho (\tau )}\}$. Then, since the system-environmnet interaction is weak in the polaron frame, we may use $\Gamma (\tau )= -\frac{\ln {s(\tau )}}{\tau }$ to find the decay rate for the strongly interacting reservoir given an arbitrary initial state, $\zeta _1\left| 0\right\rangle +\zeta _2\left| 1\right\rangle$. The detailed expression for $\Gamma (\tau )$ is given in the supplementary information.

### Modified decay rate for a strongly interacting environment

Here, we show how to work out the survival probability expressed in Eq. (7) and derive the general modified decay rate expression. In $\hbox {Tr}_{S,B}\left\{ P_{\psi }U_{S,I}^{\dagger }(\tau )U_{S,0}^{\dagger }(\tau )U_0(\tau ) U_I(\tau ) P_{\psi }\frac{e^{-\beta H_{0}}}{Z} P_{\psi } U_I^{\dagger }(\tau )U_0^{\dagger }(\tau )U_{S,0}(\tau )U_{S,I}(\tau )\right\}$, we have already evaluated $U_I(\tau ) P_{\psi }\frac{e^{-\beta H_{0}}}{Z} P_{\psi } U_I^{\dagger }(\tau )$ in Eq. (9). Moreover, we note that $U_{S,0}^{\dagger }(\tau )U_{0}(\tau )= e^{-iH_Bt}$. Now, we only need to work out the density matrix after the system evolution has been removed, that is, $U_{S,I}^{\dagger }(\tau )e^{-iH_B\tau }\Big [\rho _0 + A_1(\tau )\rho _0 + A_2(\tau )\rho _0 + \rho _0 A_1^{\dagger }(\tau ) +\rho _0 A_2^{\dagger }(\tau ) + A_1(\tau )\rho _0 A_1^{\dagger }(\tau )\Big ]e^{iH_B\tau }U_{S,I}(\tau )$. Writing $V_{S,I}(t)=e^{iH_{S,0} t}H_{I}(t)e^{-iH_{S,0} t}$, we get $V_{S,I}(t)= \frac{\Delta }{2}\sum _{\mu }(\widetilde{F}_{\mu }(t)\otimes B_{\mu })$, where $\widetilde{F}_0(t)= \sigma _{-}e^{-i\varepsilon t}$, $\widetilde{F}_1( t)= \sigma _{+}e^{i\varepsilon t}$, $B_0 = e^{\chi }$, and $B_1=e^{-\chi }$ as before. This leads to $U_{S,I}(\tau )= 1 - i\int _0^{\tau }dt_1 V_{S,I}(t_1) + (-i)^2\int _0^{{\tau }}\int _0^{t_{1}}dt_{1}dt_{2} V_{S,I}(t_{1})V_{S,I}(t_{2})+\cdots$. Using $A_S^{(1)} = - i\int _0^{\tau }dt_1 V_{S,I}(t_1)$ and $A_S^{(2)} =-\int _0^{{\tau }}\int _0^{t_{1}}dt_{1}dt_{2} V_{S,I}(t_{1})V_{S,I}(t_{2})$ now, we can conveniently write the fully time-evolved density matrix with the system evolution removed as $\rho (\tau ) = \left( 1 + A_S^{(1)\dagger }+ A_S^{(2)\dagger }\right) e^{-iH_B t}\sum _{j=1}^{N=6}T_{j}e^{iH_Bt}\left( 1 + A_S^{(1)}+ A_S^{(2)}\right)$. We work this out to second order, apply the projection operator $P_{\psi }$, and find the trace over the system and the environment in the same way as before. We then arrive at the survival probability that the modified decay rate could be found from. Details on its expression could be found in the supplementary information.

## Supplementary Information

Supplementary Information.

## Data Availability

The datasets used and/or analyzed during the current study are available from the corresponding author on reasonable request.
