# Supplementary material for: A generalized framework for the quantum Zeno and anti-Zeno effects in the strong coupling regime
Source: Sci Rep. 2022 Nov 4;12:18652. doi: 10.1038/s41598-022-23421-4 (PMC9636234; doi:10.1038/s41598-022-23421-4)
Supplement: Supplementary file 1 — Supplementary Information. [file 41598_2022_23421_MOESM1_ESM.pdf]

Supplementary information

In the Methods section, we outlined the general procedure we adopted to work out the decay rates. This supplement is meant both to expand on that procedure a little and to give explicit expressions for the effective and modified decay rates. Continuing with the notation described in the Methods section—initial state $|\psi\rangle = \zeta_1 |0\rangle + \zeta_2 |1\rangle$, normalization factor Z, the symbols C^n_{ij}, and the symbols E^n_{ij}—we find that the following works out to be the effective decay rate for an arbitrary initial state:

$$
\begin{aligned}
\Gamma(\tau) = \frac{1}{\tau}\Bigg(1 - \sum_n \mathrm{Tr}_B \Bigg\{ \frac{1}{Z}\Bigg[& |\zeta_1|^2 C^n_{00} E^n_{00} + \zeta_1\zeta_2^* e^{i\varepsilon\tau} C^n_{10} e^{\chi(\tau)} E^n_{10} + \zeta_1\zeta_2^* e^{i\varepsilon\tau} C^n_{01} e^{-\chi(t)} E^n_{01} + |\zeta_2|^2 C^n_{11} E^n_{11} \\
& + 2\,\mathrm{Re}\Bigg\{ i\frac{\Delta}{2}\int_0^\tau dt_1 |\zeta_1|^2 e^{i\varepsilon t_1} C^n_{01} E^n_{01} \widetilde{B}_0 + |\zeta_2|^2 e^{i\varepsilon t_1} C^n_{10} E^n_{10} \widetilde{B}_1 + \zeta_1\zeta_2^* e^{\chi(t)} e^{i\varepsilon(\tau - t_1)} C^n_{11} E^n_{11} \widetilde{B}_0 + \zeta_2\zeta_1^* e^{-\chi(t)} e^{-i\varepsilon(\tau-t_1)} C^n_{00} E^n_{00} \widetilde{B}_1 \Bigg\} \\
& - 2\,\mathrm{Re}\Bigg\{ \frac{\Delta^2}{4}\int_0^\tau dt_1 \int_0^{t_1} dt_2 \Bigg(|\zeta_1|^2 \Omega^{01} C^n_{00} E^n_{00} \bar{B}_1 \widetilde{B}_0 + |\zeta_2|^2 \Omega^{10} C^n_{11} E^n_{11} \bar{B}_0 \widetilde{B}_1 + \zeta_1\zeta_2^* e^{\chi(\tau)} e^{i\varepsilon\tau} \Omega^{01} C^n_{10} E^n_{10} \bar{B}_1 \widetilde{B}_0 \\
& + \zeta_2\zeta_1^* e^{-\chi(\tau)} e^{-i\varepsilon\tau} \Omega^{10} C^n_{11} E^n_{11} \bar{B}_0 \widetilde{B}_1 \Bigg) \Bigg\} + \frac{\Delta^2}{4}\int_0^\tau dt_1 \int_0^\tau dt_2 \Bigg(|\zeta_1|^2 \Omega^{10} C^n_{11} \widetilde{B}_1 E^n_{11} \bar{B}_0 + \zeta_1\zeta_2^* e^{i\varepsilon\tau} e^{\chi(\tau)} \Omega^{00} C^n_{01} \widetilde{B}_0 E^n_{01} \bar{B}_0 \\
& + \zeta_2\zeta_1^* e^{-i\varepsilon\tau} e^{-\chi(\tau)} \Omega^{11} C^n_{10} \widetilde{B}_1 E^n_{10} \bar{B}_1 + |\zeta_2|^2 \Omega^{01} C^n_{00} \widetilde{B}_0 E^n_{00} \bar{B}_1 \Bigg) \Bigg] \Bigg\} \Bigg),
\end{aligned}
\tag{1}
$$

where $\Omega^{00} = e^{-i\varepsilon(t_1+t_2)}$, $\Omega^{01} = e^{-i\varepsilon(t_1-t_2)}$, $\Omega^{10} = (\Omega^{01})^\dagger$, and $\Omega^{11} = (\Omega^{00})^\dagger$. Also, $\widetilde{B}_0 = e^{-\chi(t_1)}$ whereas $\widetilde{B}_1 = e^{\chi(t_1)}$, and $\bar{B}_0 = e^{-\chi(t_2)}$ while $\bar{B}_1 = e^{\chi(t_2)}$ with $\chi(t)$ being the time-evolved version of $\chi = \sum_k \left(\frac{2g_k}{\omega_k} b_k^\dagger - \frac{2g_k^*}{\omega_k} b_k\right)$. The b_k and $b_k^\dagger$ here are the annihilation and creation operators for the harmonic oscillator environment. Finally, $\mathrm{Tr}_B\{\ldots\}$ represents trace over the bath of harmonic oscillators. From Eq. (1), it is easy to see the bath traces that need to be evaluated to yield the correlation functions and an explicit expression for $\Gamma(\tau)$. We have, for instance, $\sum_n \mathrm{Tr}_B\left\{\frac{1}{Z}|\zeta_1|^2 C^n_{00} E^n_{00}\right\}$, $\sum_n \mathrm{Tr}_B\left\{\frac{1}{Z}|\zeta_2|^2 C^n_{11} E^n_{11}\right\}$, and so on, and to find these traces out, we use the identity $\mathrm{Tr}_B\{\rho_B e^F\} = e^{\langle F^2\rangle/2}$, where F is a linear function of the creation and annihilation operators and $\rho_B = \frac{e^{-\beta H_B}}{Z}$ with H_B being the bath Hamiltonian. The following provides a good example of the kind of bath traces we encounter:

$$
\left\langle e^{-\chi(\tau)} e^{\chi(t_1)} e^{\chi} e^{\chi(t_2)} \right\rangle_B = e^{-[\chi(\tau),\chi(t_1)]/2} e^{-[\chi(t_1),\chi]/2} e^{[\chi(t_1),\chi]/2} e^{-[\chi(t),\chi(t_2)]/2} e^{[\chi(t_1),\chi(t_2)]/2} e^{[\chi,\chi(t_2)]/2} e^{\left\langle (-\chi(\tau)+\chi(t_1)+\chi+\chi(t_2))^2/2 \right\rangle_B}.
$$

Simplifying the commutators in the exponentials above and evaluating the bath trace in $e^{\left\langle (-\chi(\tau)+\chi(t_1)+\chi+\chi(t_2))^2/2 \right\rangle_B}$, we find that this correlation function works out to be $We^{-i\Phi_I(t_2)} e^{-i\Phi_I(t_1)} e^{i\Phi_I(\tau)}$, where W is defined as

$$
W = e^{-2\Phi_{R2}} e^{-\Phi_{R1}(t_2-t_1)} e^{\Phi_{R1}(t_2-\tau)} e^{\Phi_{R1}(t_1-\tau)} e^{\Phi_{R1}(t_2)} e^{\Phi_{R1}(t_1)} e^{-\Phi_{R1}(\tau)} e^{i\Phi_I(t_2-t_1)} e^{-i\Phi_I(t_2-\tau)} e^{i\Phi_I(t_1-\tau)}.
$$

A very similar expression shows up in other bath traces as well, and we denote it by

$$
W' = e^{-2\Phi_{R2}} e^{\Phi_{R1}(t_2-t_1)} e^{-\Phi_{R1}(t_2-\tau)} e^{\Phi_{R1}(t_1-\tau)} e^{\Phi_{R1}(t_2)} e^{-\Phi_{R1}(t_1)} e^{\Phi_{R1}(\tau)} e^{-i\Phi_I(t_2-t_1)} e^{i\Phi_I(t_2-\tau)} e^{-i\Phi_I(t_1-\tau)}.
$$

In W and W', $\Phi_{R1} = 4\int_0^\infty d\omega J(\omega) \frac{\cos(\omega\tau)}{\omega^2} \coth\left(\frac{\beta\omega}{2}\right)$, $\Phi_{R2} = 4\int_0^\infty d\omega J(\omega)(\coth\left(\frac{\beta\omega}{2}\right)/\omega^2)$, and $\Phi_I = 4\int_0^\infty d\omega J(\omega)\frac{\sin(\omega\tau)}{\omega^2}$, where the environment spectral densities have been introduced as $\sum_k |g_k|^2(\cdots) \to \int_0^\infty d\omega J(\omega)(\cdots)$. If we now use W and W' to

continue doing the bath traces, we find that the effective decay rate is explicitly given by

$$
\begin{aligned}
\Gamma(\tau) = \frac{1}{\tau}\Bigg\{ 1 - \frac{1}{Z_s}\Bigg[& |\zeta_1|^6 e^{-\beta\varepsilon/2} + |\zeta_1|^4|\zeta_2|^2 e^{\beta\varepsilon/2} + |\zeta_1|^2|\zeta_2|^4 e^{-\beta\varepsilon/2} + |\zeta_2|^6 e^{\beta\varepsilon/2} \\
& + 2\,\mathrm{Re}\Bigg\{ |\zeta_1|^2|\zeta_2|^4 e^{i\varepsilon\tau} e^{\beta\varepsilon/2} e^{-\Phi_C(\tau)} + |\zeta_1|^4|\zeta_2|^2 e^{i\varepsilon\tau} e^{-\beta\varepsilon/2} e^{-\Phi_C^*(\tau)} \Bigg\} \\
& + 2\,\mathrm{Re}\Bigg\{ i\frac{\Delta}{2}\int_0^\tau dt_1 \Bigg(|\zeta_1|^4 \zeta_1\zeta_2^* e^{-i\varepsilon t_1} e^{-\beta\varepsilon/2} e^{-\Phi_C(t_1)} + |\zeta_1\zeta_2|^2 \zeta_1\zeta_2^* e^{-i\varepsilon t_1} e^{\beta\varepsilon/2} e^{-\Phi_C^*(t_1)} \\
& + |\zeta_1\zeta_2|^2 \zeta_2\zeta_1^* e^{i\varepsilon t_1} e^{-\beta\varepsilon/2} e^{-\Phi_C^*(t_1)} + |\zeta_2|^4 \zeta_2\zeta_1^* e^{i\varepsilon t_1} e^{\beta\varepsilon/2} e^{-\Phi_C(t_1)} \\
& + |\zeta_2|^4 \zeta_1\zeta_2^* e^{i\varepsilon\tau} e^{-i\varepsilon t_1} e^{\beta\varepsilon/2} e^{-\Phi_C^*(t_1-\tau)} + |\zeta_1\zeta_2|^2 \zeta_1\zeta_2^* e^{i\varepsilon\tau} e^{-i\varepsilon t_1} e^{-\beta\varepsilon/2} e^{-\Phi_C^*(t_1-\tau)} e^{-2i\Phi_I(\tau)} e^{2i\Phi_I(t_1)} \\
& + |\zeta_1|^4 \zeta_2\zeta_1^* e^{-i\varepsilon\tau} e^{i\varepsilon t_1} e^{-\beta\varepsilon/2} e^{-\Phi_C^*(t_1-\tau)} + |\zeta_1\zeta_2|^2 \zeta_2\zeta_1^* e^{-i\varepsilon\tau} e^{i\varepsilon t_1} e^{\beta\varepsilon/2} e^{-\Phi_C^*(t_1-\tau)} e^{-2i\Phi_I(\tau)} e^{2i\Phi_I(t_1)} \Bigg) \Bigg\} \\
& - 2\,\mathrm{Re}\Bigg\{ \frac{\Delta^2}{4}\int_0^\tau dt_1 \int_0^{t_1} dt_2 \Bigg(|\zeta_1|^6 e^{-i\varepsilon t_1} e^{i\varepsilon t_2} e^{-\beta\varepsilon/2} e^{-\Phi_C^*(t_2-t_1)} \\
& + |\zeta_1|^4|\zeta_2|^2 e^{-i\varepsilon t_1} e^{i\varepsilon t_2} e^{\beta\varepsilon/2} e^{-\Phi_C^*(t_2-t_1)} e^{2i\Phi_I(t_2)} e^{-2i\Phi_I(t_1)} \\
& + |\zeta_1|^2|\zeta_1\zeta_2|^2 e^{i\varepsilon t_1} e^{-i\varepsilon t_2} e^{-\beta\varepsilon/2} e^{-\Phi_C^*(t_2-t_1)} e^{2i\Phi_I(t_2)} e^{-2i\Phi_I(t_1)} \\
& + |\zeta_2|^6 e^{i\varepsilon t_1} e^{-i\varepsilon t_2} e^{\beta\varepsilon/2} e^{-\Phi_C^*(t_2-t_1)} + |\zeta_1\zeta_2|^2|\zeta_1|^2 e^{i\varepsilon\tau} e^{-i\varepsilon t_1} e^{i\varepsilon t_2} e^{-\beta\varepsilon/2} W' e^{-i\Phi_I(t_2)} e^{i\Phi_I(t_1)} e^{-i\Phi_I(\tau)} \\
& + |\zeta_1\zeta_2|^2|\zeta_2|^2 e^{i\varepsilon\tau} e^{-i\varepsilon t_1} e^{i\varepsilon t_2} e^{\beta\varepsilon/2} W' e^{i\Phi_I(t_2)} e^{-i\Phi_I(t_1)} e^{i\Phi_I(\tau)} + |\zeta_1\zeta_2|^2|\zeta_1|^2 e^{-i\varepsilon\tau} e^{i\varepsilon t_1} e^{-i\varepsilon t_2} e^{-\beta\varepsilon/2} W' e^{i\Phi_I(t_2)} e^{-i\Phi_I(t_1)} e^{i\Phi_I(\tau)} \\
& + |\zeta_1\zeta_2|^2|\zeta_1|^2 e^{-i\varepsilon\tau} e^{i\varepsilon t_1} e^{-i\varepsilon t_2} e^{\beta\varepsilon/2} W' e^{-i\Phi_I(t_2)} e^{i\Phi_I(t_1)} e^{-i\Phi_I(\tau)} \Bigg) \Bigg\} \\
& + \frac{\Delta^2}{4}\int_0^\tau dt_1 \int_0^{\tau} dt_2 \Bigg(|\zeta_1\zeta_2|^2|\zeta_1|^2 e^{i\varepsilon t_1} e^{-i\varepsilon t_2} e^{-\beta\varepsilon/2} e^{-\Phi_C^*(t_2-t_1)} e^{2i\Phi_I(t_2)} e^{-2i\Phi_I(t_1)} \\
& + |\zeta_1\zeta_2|^2|\zeta_2|^2 e^{i\varepsilon t_1} e^{-i\varepsilon t_2} e^{\beta\varepsilon/2} e^{-\Phi_C^*(t_2-t_1)} + |\zeta_1\zeta_2|^2|\zeta_1|^2 e^{-i\varepsilon t_1} e^{i\varepsilon t_2} e^{-\beta\varepsilon/2} e^{-\Phi_C^*(t_2-t_1)} \\
& + |\zeta_1\zeta_2|^2|\zeta_2|^2 e^{-i\varepsilon t_1} e^{i\varepsilon t_2} e^{\beta\varepsilon/2} e^{-\Phi_C^*(t_2-t_1)} e^{2i\Phi_I(t_2)} e^{-2i\Phi_I(t_1)} \\
& + |\zeta_1|^2 \zeta_1^2 \zeta_2^{*2} e^{i\varepsilon\tau} e^{-i\varepsilon t_1} e^{-i\varepsilon t_2} e^{-\beta\varepsilon/2} W e^{i\Phi_I(t_2)} e^{i\Phi_I(t_1)} e^{-i\Phi_I(\tau)} + |\zeta_2|^2 \zeta_1^2 \zeta_2^{*2} e^{i\varepsilon\tau} e^{-i\varepsilon t_1} e^{-i\varepsilon t_2} e^{\beta\varepsilon/2} W e^{-i\Phi_I(t_2)} e^{-i\Phi_I(t_1)} e^{i\Phi_I(\tau)} \\
& + |\zeta_1|^2 \zeta_2^2 \zeta_1^{*2} e^{-i\varepsilon\tau} e^{i\varepsilon t_1} e^{i\varepsilon t_2} e^{-\beta\varepsilon/2} W e^{-i\Phi_I(t_2)} e^{-i\Phi_I(t_1)} e^{i\Phi_I(\tau)} + |\zeta_2|^2 \zeta_2^2 \zeta_1^{*2} e^{-i\varepsilon\tau} e^{i\varepsilon t_1} e^{i\varepsilon t_2} e^{\beta\varepsilon/2} W e^{i\Phi_I(t_2)} e^{i\Phi_I(t_1)} e^{-i\Phi_I(\tau)} \Bigg) \Bigg] \Bigg\}.
\end{aligned}
\tag{2}
$$

Z_s here is what is left of Z after the bath traces have been done, and it turns out that $Z_s = |\zeta_1|^4 e^{-\beta\varepsilon/2} + |\zeta_1\zeta_2|^2 e^{\beta\varepsilon/2} + +|\zeta_1\zeta_2|^2 e^{-\beta\varepsilon/2} + |\zeta_2|^4 e^{\beta\varepsilon/2}$. $\Phi_C(t) = \Phi_R(t) - i\Phi_I(t)$ with $\Phi_R = 4\int_0^\infty d\omega J(\omega)\frac{1-\cos(\omega\tau)}{\omega^2}\coth\left(\frac{\beta\omega}{2}\right)$, and Φ_I is the same as before. We model the spectral density as $J(\omega) = G\omega^s\omega_c^{1-s}e^{-\omega/\omega_c}$, where G is a dimensionless parameter characterizing the strength of the system-environment coupling, ω_c is the cut-off frequency, and s is the Ohmicity parameter. Setting $s = 2$, which corresponds to a super-Ohmic spectral density, yields $\Phi_R = 4G\left(1 - \frac{1}{1+\omega_c^2 t^2}\right)$, $\Phi_{R1} = \frac{4G}{1+\omega_c^2 t^2}$, $\Phi_{R2} = 4G$, and $\Phi_I = \frac{4Gt}{\omega_c\left(\frac{1}{\omega_c^2}+t^2\right)}$.

Now that we explicitly have the effective decay rate at hand, we proceed to similarly finding the modified decay rate. In the Methods section, the latter was defined as the decay rate that is obtained after the removal of system evolution. More precisely, we remove the evolution due to the central quantum system from the fully time-evolved system-environment density matrix before it is used to calculate the survival probability and the decay rate. Using time-dependent perturbation theory to do this removal then, we find, as explained in the paper as well, that the modified decay rate is $\Gamma_n(\tau) = \Gamma(\tau) + \Gamma_{\mathrm{mod}}(\tau)$, where $\Gamma(\tau)$ is simply the effective decay rate in Eq. (2) and $\Gamma_{\mathrm{mod}}(\tau)$ is comprised of some extra terms like those in the trace in Eq. (1). Consequently, in order to work out $\Gamma_n(\tau)$ explicitly, we need to calculate some additional bath traces. We find that the modified decay rate is

$$
\Gamma_n(\tau) = \Gamma(\tau) - \frac{1}{\tau}\left[\frac{1}{Z_s}\left(N_1 + N_2 + N_3 + N_4 + N_5\right)\right], \tag{3}
$$

where

$$
\begin{aligned}
N_1 = &-i\frac{\Delta}{2}\int_0^\tau dt_1 \Bigg[|\zeta_1|^2\zeta_1\zeta_2^* e^{-i\varepsilon t_1}\left(|\zeta_1|^2 e^{-\beta\varepsilon/2}e^{-\Phi_C(\tau)}+|\zeta_2|^2 e^{\beta\varepsilon/2}e^{-\Phi_C^*(\tau)}\right)+|\zeta_2|^2\zeta_1\zeta_2^* e^{-i\varepsilon t_1}\left(|\zeta_1|^2 e^{-\beta\varepsilon/2}+|\zeta_2|^2 e^{\beta\varepsilon/2}\right)\\
&+|\zeta_1|^2\zeta_2\zeta_1^* e^{i\varepsilon t_1}\left(|\zeta_1|^2 e^{-\beta\varepsilon/2}+|\zeta_2|^2 e^{\beta\varepsilon/2}\right)+|\zeta_2|^2\zeta_2\zeta_1^* e^{i\varepsilon t_1}\left(|\zeta_1|^2 e^{-\beta\varepsilon/2}e^{-\Phi_C^*(\tau)}+|\zeta_2|^2 e^{\beta\varepsilon/2}e^{-\Phi_C(\tau)}\right)\Bigg]\\
&+\frac{\Delta^2}{4}\int_0^\tau dt_1\int_0^\tau dt_2\Bigg[|\zeta_1|^4 e^{i\varepsilon t_1}e^{-i\varepsilon t_2}\left(|\zeta_1|^2 e^{-\beta\varepsilon/2}e^{-\Phi_C(t_1-\tau)}+|\zeta_2|^2 e^{\beta\varepsilon/2}e^{-\Phi_C^*(t_1-\tau)}e^{2i\Phi_I(t_1)}e^{-2i\Phi_I(\tau)}\right)\\
&+|\zeta_1\zeta_2|^2 e^{i\varepsilon t_1}e^{-i\varepsilon t_2}\left(|\zeta_1|^2 e^{-\beta\varepsilon/2}e^{-\Phi_C^*(t_1)}+|\zeta_2|^2 e^{\beta\varepsilon/2}e^{-\Phi_C(t_1)}\right)\\
&+|\zeta_1\zeta_2|^2 e^{-i\varepsilon t_1}e^{i\varepsilon t_2}\left(|\zeta_1|^2 e^{-\beta\varepsilon/2}e^{-\Phi_C(t_1)}+|\zeta_2|^2 e^{\beta\varepsilon/2}e^{-\Phi_C^*(t_1)}\right)\\
&+|\zeta_2|^4 e^{-i\varepsilon t_1}e^{i\varepsilon t_2}\left(|\zeta_1|^2 e^{-\beta\varepsilon/2}e^{-\Phi_C^*(t_1-\tau)}e^{2i\Phi_I(t_1)}e^{-2i\Phi_I(\tau)}+|\zeta_2|^2 e^{\beta\varepsilon/2}e^{-\Phi_C^*(t_1-\tau)}\right)\\
&+|\zeta_1\zeta_2|^2 e^{i\varepsilon t_1}e^{-i\varepsilon t_2}\left(|\zeta_1|^2 e^{-\beta\varepsilon/2}e^{-\Phi_C^*(\tau-t_1)}e^{2i\Phi_I(\tau)}e^{-2i\Phi_I(t_1)}+|\zeta_2|^2 e^{\beta\varepsilon/2}e^{-\Phi_C(\tau-t_1)}\right)\\
&+(\zeta_1\zeta_2^*)^2 e^{-i\varepsilon t_1}e^{-i\varepsilon t_2}\left(|\zeta_1|^2 e^{-\beta\varepsilon/2}e^{-\Phi_C(t_1)}+|\zeta_2|^2 e^{\beta\varepsilon/2}e^{-\Phi_C^*(t_1)}\right)\\
&+(\zeta_2\zeta_1^*)^2 e^{i\varepsilon t_1}e^{i\varepsilon t_2}\left(|\zeta_1|^2 e^{-\beta\varepsilon/2}e^{-\Phi_C^*(t_1)}+|\zeta_2|^2 e^{\beta\varepsilon/2}e^{-\Phi_C(t_1)}\right)\\
&+|\zeta_1\zeta_2|^2 e^{-i\varepsilon t_1}e^{i\varepsilon t_2}\left(|\zeta_1|^2 e^{-\beta\varepsilon/2}e^{-\Phi_C(t_1-\tau)}+|\zeta_2|^2 e^{\beta\varepsilon/2}e^{-\Phi_C^*(t_1-\tau)}e^{2i\Phi_I(\tau)}e^{-2i\Phi_I(t_1)}\right)\Bigg],
\end{aligned}
$$

$$
\begin{aligned}
N_2 = &-\frac{\Delta^2}{4}\int_0^\tau dt_1\int_0^{t_1} dt_2\Bigg[|\zeta_1|^4 e^{i\varepsilon t_1}e^{-i\varepsilon t_2}\left(|\zeta_1|^2 e^{-\beta\varepsilon/2}+|\zeta_2|^2 e^{\beta\varepsilon/2}\right)+|\zeta_1\zeta_2|^2 e^{i\varepsilon t_1}e^{-i\varepsilon t_2}\left(|\zeta_1|^2 e^{-\beta\varepsilon/2}e^{-\Phi_C^*(\tau)}+|\zeta_2|^2 e^{\beta\varepsilon/2}e^{-\Phi_C(\tau)}\right)\\
&+|\zeta_1\zeta_2|^2 e^{-i\varepsilon t_1}e^{i\varepsilon t_2}\left(|\zeta_1|^2 e^{-\beta\varepsilon/2}e^{-\Phi_C(\tau)}+|\zeta_2|^2 e^{\beta\varepsilon/2}e^{-\Phi_C^*(\tau)}\right)+|\zeta_2|^4 e^{-i\varepsilon t_1}e^{i\varepsilon t_2}\left(|\zeta_1|^2 e^{-\beta\varepsilon/2}+|\zeta_2|^2 e^{\beta\varepsilon/2}\right)\Bigg],
\end{aligned}
$$

$$
\begin{aligned}
N_3 = &\,i\frac{\Delta}{2}\int_0^\tau dt_1\Bigg[|\zeta_1|^2\zeta_2\zeta_1^* e^{i\varepsilon t_1}\left(|\zeta_1|^2 e^{-\beta\varepsilon/2}e^{-\Phi_C^*(\tau)}+|\zeta_2|^2 e^{-\beta\varepsilon/2}e^{-\Phi_C(\tau)}\right)+|\zeta_1|^2\zeta_1\zeta_2^* e^{-i\varepsilon t_1}\left(|\zeta_1|^2 e^{-\beta\varepsilon/2}+|\zeta_2|^2 e^{\beta\varepsilon/2}\right)\\
&+\zeta_2\zeta_1^*|\zeta_2|^2 e^{i\varepsilon t_1}\left(|\zeta_1|^2 e^{-\beta\varepsilon/2}+|\zeta_2|^2 e^{\beta\varepsilon/2}\right)+|\zeta_2|^2\zeta_1\zeta_2^* e^{-i\varepsilon t_1}\left(|\zeta_1|^2 e^{-\beta\varepsilon/2}e^{-\Phi_C(\tau)}+|\zeta_2|^2 e^{\beta\varepsilon/2}e^{-\Phi_C^*(\tau)}\right)\Bigg]\\
&-\frac{\Delta^2}{4}\int_0^\tau dt_1\int_0^\tau dt_2\Bigg[|\zeta_1\zeta_2|^2 e^{-i\varepsilon t_1}e^{i\varepsilon t_2}\left(|\zeta_1|^2 e^{-\beta\varepsilon/2}e^{-\Phi_C^*(t_1-\tau)}e^{2i\Phi_I(t_1)}e^{-2i\Phi_I(\tau)}+|\zeta_2|^2 e^{\beta\varepsilon/2}e^{-\Phi_C^*(t_1-\tau)}\right)\\
&+(\zeta_1\zeta_2^*)^2 e^{-i\varepsilon t_1}e^{-i\varepsilon t_2}\left(|\zeta_1|^2 e^{-\beta\varepsilon/2}e^{-\Phi_C(t_1)}+|\zeta_2|^2 e^{\beta\varepsilon/2}e^{-\Phi_C^*(t_1)}\right)\\
&+(\zeta_2\zeta_1^*)^2 e^{i\varepsilon t_1}e^{i\varepsilon t_2}\left(|\zeta_1|^2 e^{-\beta\varepsilon/2}e^{-\Phi_R^{(}t_1-\tau)}e^{-i\Phi_I(t_1)}+|\zeta_2|^2 e^{\beta\varepsilon/2}e^{-\Phi_R(t_1-\tau)}e^{i\Phi_I(t_1)}\right)\\
&+|\zeta_1\zeta_2|^2 e^{i\varepsilon t_1}e^{-i\varepsilon t_2}\left(|\zeta_1|^2 e^{-\beta\varepsilon/2}e^{-\Phi_C^*(t_1-\tau)}+|\zeta_2|^2 e^{\beta\varepsilon/2}e^{-\Phi_C^*(t_1-\tau)}e^{2i\Phi_I(t_1)}e^{-2i\Phi_I(\tau)}\right)\\
&-|\zeta_1|^4 e^{-i\varepsilon t_1}e^{i\varepsilon t_2}\left(|\zeta_1|^2 e^{-\beta\varepsilon/2}e^{-\Phi_C(t_1-\tau)}+|\zeta_2|^2 e^{\beta\varepsilon/2}e^{-\Phi_C^*(t_1-\tau)}e^{-2i\Phi_I(t_1)}e^{2i\Phi_I(\tau)}\right)\\
&-|\zeta_1\zeta_2|^2 e^{i\varepsilon t_1}e^{-i\varepsilon t_2}\left(|\zeta_1|^2 e^{-\beta\varepsilon/2}e^{-\Phi_C^*(t_1)}+|\zeta_2|^2 e^{\beta\varepsilon/2}e^{-\Phi_C(t_1)}\right)\\
&-|\zeta_1\zeta_2|^2 e^{i\varepsilon t_1}e^{-i\varepsilon t_2}\left(|\zeta_1|^2 e^{-\beta\varepsilon/2}e^{-\Phi_C(t_1)}+|\zeta_2|^2 e^{\beta\varepsilon/2}e^{-\Phi_C^*(t_1)}\right)\\
&-|\zeta_2|^4 e^{i\varepsilon t_1}e^{-i\varepsilon t_2}\left(|\zeta_1|^2 e^{-\beta\varepsilon/2}e^{-\Phi_C^*(\tau-t_1)}e^{-2i\Phi_I(t_1)}e^{2i\Phi_I(\tau)}+|\zeta_2|^2 e^{\beta\varepsilon/2}e^{-\Phi_C(t_1-\tau)}\right)\Bigg],
\end{aligned}
$$

$$
\begin{aligned}
N_4 = &\,\frac{\Delta^2}{4}\int_0^\tau dt_1\int_0^\tau dt_2\Bigg[|\zeta_1\zeta_2|^2 e^{i\varepsilon t_1}e^{-i\varepsilon t_2}\left(|\zeta_1|^2 e^{-\beta\varepsilon/2}+|\zeta_2|^2 e^{\beta\varepsilon/2}\right)+(\zeta_1\zeta_2^*)^2 e^{-i\varepsilon t_1}e^{-i\varepsilon t_2}\left(e^{-\beta\varepsilon/2}e^{-\Phi_C(\tau)}+e^{\beta\varepsilon/2}e^{-\Phi_C^*(\tau)}\right)\\
&+(\zeta_2\zeta_1^*)^2 e^{i\varepsilon t_1}e^{i\varepsilon t_2}\left(e^{-\beta\varepsilon/2}e^{-\Phi_C^*(\tau)}+e^{\beta\varepsilon/2}e^{-\Phi_C(\tau)}\right)+|\zeta_2\zeta_1|^2 e^{-i\varepsilon t_1}e^{i\varepsilon t_2}\left(e^{-\beta\varepsilon/2}+e^{\beta\varepsilon/2}\right)\Bigg],
\end{aligned}
$$

$$N_5 = -\frac{\Delta^2}{4}\int_0^\tau dt_1 \int_0^\tau dt_2 \Big[|\zeta_1|^2 e^{-i\varepsilon t_1} e^{i\varepsilon t_2} \left(|\zeta_1|^4 e^{-\beta\varepsilon/2} + |\zeta_1\zeta_2|^2 e^{\beta\varepsilon/2} \right) + |\zeta_1\zeta_2|^2 e^{i\varepsilon t_1} e^{-i\varepsilon t_2} \left(|\zeta_1|^2 e^{-\beta\varepsilon/2} e^{-\Phi_C^*(\tau)} + |\zeta_2|^2 e^{\beta\varepsilon/2} e^{-\Phi_C(\tau)} \right)$$
$$+ |\zeta_1\zeta_2|^2 e^{-i\varepsilon t_1} e^{i\varepsilon t_2} \left(|\zeta_1|^2 e^{-\beta\varepsilon/2} e^{-\Phi_C(\tau)} + |\zeta_2|^2 e^{\beta\varepsilon/2} e^{-\Phi_C^*(\tau)} \right) + |\zeta_2|^2 e^{i\varepsilon t_1} e^{-i\varepsilon t_2} \left(|\zeta_1\zeta_2|^2 e^{-\beta\varepsilon/2} + |\zeta_2|^4 e^{\beta\varepsilon/2} \right) \Big],$$

and Z_s is the same as described for the effective decay rate. This completes the goal that we set out in this supplement with: giving explicit expressions for both the effective and the modified decay rates. Something worth remembering is that all these calculations are correct to second order only because we used time-dependent perturbation theory to second order. Also note that in the calculation of the effective and the modified decay rates, we did not require that the temperature be zero (or that $\beta \to \infty$), for we were deriving the most general expressions. We work at zero temperature only for the plots we present in the paper just for the sake of simplicity.
